# The complete plastid genome of Hong Kong Orchid Tree, *Bauhinia* × *blakeana* Dunn (Leguminosae)

**DOI:** 10.1080/23802359.2019.1674218

**Published:** 2019-10-09

**Authors:** Shiran Gu, Qiang Lai, Qiubiao Zeng, Tieyao Tu, Dianxiang Zhang

**Affiliations:** a Key Laboratory of Plant Resources Conservation and Sustainable Utilization, South China Botanical Garden, Chinese Academy of Sciences, Guangzhou, China;; b University of Chinese Academy of Sciences, Beijing, China;; c Guangxi University, Nanning, China

**Keywords:** *Bauhinia*, chloroplast genome, Fabaceae, legume family, phylogeny

## Abstract

*Bauhinia* × *blakeana* Dunn, or the Hong Kong Orchid Tree, is a popular ornamental plant in the tropical and subtropical regions. In this study, we report and characterise the complete plastid genome of *B.* × *blakeana* in an effort to provide genomic resources for genetic utilization. The complete plastome is 156,100 bp in length and contains the typical quadripartite structure, including two inverted repeat (IR) regions of 25,847 bp, a large single-copy (LSC) region of 86,484 bp and a small single-copy (SSC) region of 17,922 bp. 126 genes are annotated, including 81 protein-coding genes, 37 transfer RNA genes, and eight ribosomal RNA genes. The phylogenetic analysis based on the plastomes from *B.* × *blakeana* and 11 previously reported species of Cercidoideae suggested a sister-relationship between *B.* × *blakeana* and *B. acuminata* L. with strong bootstrap support.

*Bauhinia* × *blakeana* Dunn, or the Hong Kong Orchid Tree, is a well-known ornamental tree of the legume family in tropical and subtropical region. The bilobed leaves of *B.* × *blakeana* are butterfly-like, and its beautiful flowers are large, fragrant, and orchid-like, leading Hong Kong and Ilan of Taiwan choose it as a city flower. It was first described under the name *B. blakeana* by Dunn in 1908 based on living trees from Hong Kong (Dunn 1908). However, this plant is often sterile and evidence of morphology and Sanger sequencing suggests that it could be a hybrid of *B. purpurea* L. and *B. variegata* L. (Carol et al. 2005; Luo et al. 2006; Mak et al. 2008; Sinha and Singh 2013). To better understand the plastid genome characterisation of this plant, we generated the complete plastid genome of the species using the method of genome skimming.

The fresh leaf tissues were collected from South China Agricultural University, Guangzhou, China (113.35°E, 23.16°N). Voucher specimens (HN1904243_3) were deposited in the herbarium of South China Botanical Garden (IBSC). We isolated the whole genomic DNA by a modified CTAB method (Doyle and Doyle 1987). We fragmented the isolated total genomic DNA into 300–500 bp in length to construct a library following the manufacturer’s manual (Illumina). Paired-end (PE) sequencing was conducted on the Illumina HiSeq X-Ten instrument at Beijing Genomics Institute (BGI) in Wuhan, China. We used GetOrganelle pipeline (Bankevich et al. 2012; Langmead and Salzberg 2012; Wick et al. 2015; Jin et al. 2018) to assemble the plastome. We employed Plastid Genome Annotator (PGA) (Qu et al. 2019) and Geneious (Kearse et al. 2012) to verify the accuracy of the assembly and to annotate the plastome. The annotated plastome has been deposited in GenBank (accession number: MN413506).

To reconstruct the phylogenetic position of *Bauhinia* × *blakeana*, we included 12 plastid genomes in previous publications and unpublished data in GenBank (Figure 1) (Sabir et al. 2014; Wang et al. 2017 , 2018). We aligned the data matrix using MAFFT (Katoh and Standley 2013) with default parameters. The maximum likelihood tree was built in IQ-tree (Trifinopoulos et al. 2016) using models recommended by ModelFinder (Kalyaanamoorthy et al. 2017) based on a data matrix which contains 77 CDS sequences. The branch supports were estimated using 1000 interations of bootstrap.

**Figure 1.**
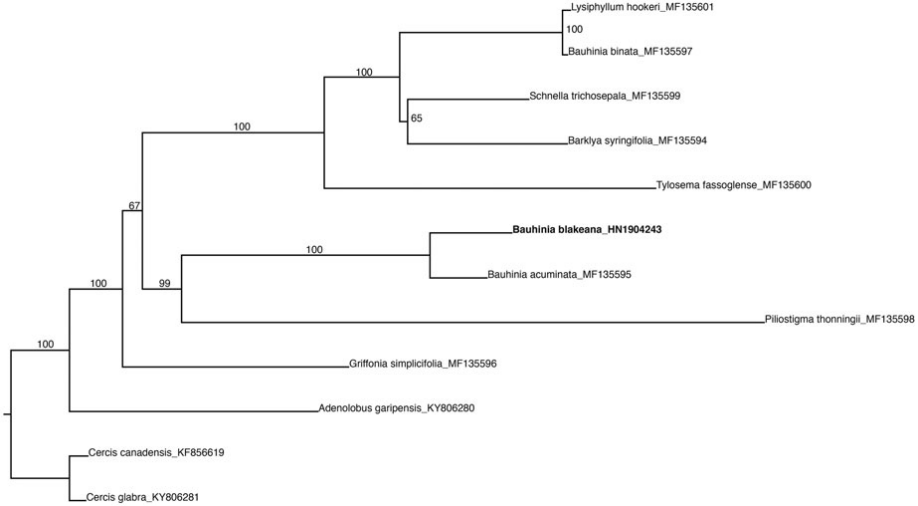
The maximum-likelihood (ML) phylogenetic tree based on the concatenation of 77 protein-coding sequences. Numbers on the branches are bootstrap support values based on 1000 replicates.

The complete plastid genome of *Bauhinia* × *blakeana* was 156,100 bp in length and showed a typical quadripartite structure: a large single copy (LSC) region of 86,484 bp and a small single copy (SSC) region of 17,922 bp, respectively. These two regions were separated by two inverted repeat regions (IRa and IRb), each of 25,847 bp in length. A total of 126 functional genes were recovered, consisting of 81 protein-coding genes, 37 tRNA genes and eight rRNA genes. The overall GC content was 36.4%. The phylogenetic analysis suggested that *B.* × *blakeana* and *B. acuminata* L. form a strongly supported clade, which is sister to *Piliostigma thonningii* (Schum.) Milne-Redh and other nine species of subfamily Cercidoideae with strong bootstrap support (Figure 1). The plastid genome of *B.* × *blakeana* reported here could be helpful for answering questions regarding its hybrid origin and genetic diversity in future when comparable data of more related species are available, especially the putative parental species of *B. purpurea* and *B. variegata*.
